# New Perspectives on Endoscopic Management of Liver and Pancreatic Cancer

**DOI:** 10.3390/cancers15051549

**Published:** 2023-03-01

**Authors:** Barbara Lattanzi, Daryl Ramai, Maura Galentino, Beatrice Martino, Antonio Facciorusso

**Affiliations:** 1 Gastroenterology and Emergency Endoscopy Unit, Department of Emergency, Sandro Pertini Hospital of Rome, 00157 Rome, Italy; 2 Gastroenterology & Hepatology, University of Utah Health, Salt Lake City, UT 84044, USA; 3 Section of Gastroenterology, Department of Medical Sciences, University of Foggia, 71122 Foggia, Italy

Liver and pancreatic cancers are major health issues which represent a clinical and economic burden worldwide. Hepatocellular carcinoma (HCC) arises from the primary cells of the liver and is the third most common cause of cancer-related death as well as the leading cause of mortality in cirrhotic patients [1]. Cholangiocarcinoma (CCA) is the second most frequent primary liver malignancy and arises from the epithelial cells lining the biliary tree. This cancer exhibits a poor prognosis and resistance to chemotherapeutic agents [2]. Pancreatic ductal adenocarcinoma (PDAC) is the most prevalent neoplastic disease of the pancreas and represents the fourth leading cause of cancer-related mortality [2]. It is projected to become the second leading cause of cancer-related mortality by 2030 [3].

Due to their high incidence rate and aggressive nature, an early diagnosis of these tumors is very important to ensuring improved survival outcomes [4,5,6,7]. Improvements in diagnostic tools and therapeutic strategies have led to consistent progress in the life expectancy of patients affected by these neoplasms. Nevertheless, a considerable proportion of patients present with advanced tumors or metastases which are not suitable for curative treatments. 

Endoscopy, in particular endoscopic ultrasound (EUS) and endoscopic retrograde cholangio-pancreatography (ERCP), represent valuable tools for the evaluation and diagnosis of pancreatic and several gastrointestinal tumors [8,9]. Moreover, endoscopy might represent a useful therapeutic option for selected patients [10]. In this Special Issue, we present five original papers which demonstrates the diagnostic and therapeutic uses of endoscopy for liver and pancreatic cancers.

Concerning diagnostic tools, liver biopsy (LB) represents the gold standard method for the definitive diagnosis of focal and parenchymal diseases of the liver. Traditionally, LB has been commonly performed using a percutaneous approach (PC-LB) (under CT scan or ultrasonographic guidance) or through a transjugular (TJ) approach. In recent years, endoscopic ultrasound (EUS)-guided liver biopsy has been adopted as a good alternative to PC and TJ approaches [11]. Facciorusso et al. compared PC-LB and EUS-LB methods in terms of diagnostic outcomes including accuracy and safety for both focal and parenchymal liver diseases [12]. Fifty-four patients undergoing endoscopic ultrasound for different primary EUS indications, who also benefited from a liver biopsy, were compared to 62 patients who underwent PC-LB. The results did not show any statistically significant difference in terms of diagnostic adequacy or accuracy, and no differences in severe adverse events were observed in both groups. On the other hand, the total sample length appeared to be greater in the PC-LB group and EUS-LB was associated with a significantly longer procedure time. Thus, the authors concluded that there was no evidence to support the widespread use of EUS-LB except in cases where it is used in conjunction with other endoscopic procedures. 

Within the biliary tract, differentiating between benign and malignant biliary stenosis (BS) is challenging, and tissue diagnosis plays a crucial role. The paper by Troncone et al. aimed at evaluating the diagnostic yield of EUS-guided fine-needle aspiration (FNA)/ fine-needle biopsy (FNB) plus ERCP with brushing or forceps biopsy in BS [13]. The authors analyzed 47 patients with BS who underwent endoscopic procedures. Patients with BS secondary to pancreatic cancer were excluded to analyze data relating to a more homogeneous population. They concluded that the combination of EUS and ERCP tissue sampling increased diagnostic accuracy in defining the etiology of BS. Moreover, repeating EUS or ERCP-based tissue sampling increased the diagnostic potential of both procedures; thus, reducing the risk of referring non-malignant cases for surgery. 

Concerning pancreatic tumors, EUS FNA/B represents the most accurate method of diagnosing these lesions; nevertheless, this technique is not without its flaws. In this Special Issue, Togliani and colleagues aimed to identify factors that impaired EUS tissue sampling diagnostic adequacy [14]. In this paper, the authors analyzed the performance of tissue acquisition in 316 EUS-FNA and 91 EUS-FNB patients with a suspicion of having pancreatic neoplasms. A four-level score measuring the degree of adequacy and a score for the grade of fibrosis were applied in this study. The authors found that the adequacy of pancreatic EUS tissue acquisition was directly correlated with the performance of three or more needle passes and the cell block, regardless of the type of needle used. Moreover, sample adequacy was negatively affected by the location of the lesion in the head/uncinate process; this result is probably due to the finding of a higher grade of tissue fibrosis in lesions located in the head/uncinate process [1].

Following the widespread adoption of EUS for pancreatic tissue acquisition in recent decades, prior reports on the risk of seeding after EUS appears to be lower when compared to the percutaneous route [15]. However, concerns on this topic still lingered. Recently, a systematic review and meta-analysis determined that the incidence of pancreatic adenocarcinoma needle-tract seeding was estimated at 0.4%, albeit without an apparent impact on prognosis [16]. However, little is known about the natural history of this disease and its management. In this scenario, Archibugi et al. elaborated through a systematic review case of needle-tract seeding and analyzed their management and outcomes [17]. The authors reported 46 cases in which PDAC seeding on the needle track was deemed extremely likely. Most cases were in the gastric wall. The data shows that this is a rare complication and usually occurs late (median 19 months from EUS-FNA). Furthermore, this phenomenon should be distinguished from “typical” distant disease recurrence, since, when treated more aggressively with repeated surgery, the overall survival seems longer compared to that of patients treated with palliative treatments.

Lastly, endoscopy might represent a useful therapeutic instrument in patients affected by locally advanced PDAC. Several ablative therapies have been proposed and tested in clinical practice [18,19]. However, the lack of definitive evidence for a survival advantage as well as its high costs represents barriers for adoption and requires additional studies. Testoni et al. contributed to this Special Issue with a phase II randomized controlled trial aimed at investigating the efficacy of thermal ablation with the HybridTherm probe under EUS guidance as a complement to chemotherapy versus standard chemotherapy alone, in locally advanced and borderline resectable PDAC [20]. A total of 17 and 20 patients were randomized in combined and chemotherapy-alone arms, respectively. The authors found an improved 6-month progression-free survival rate in the combined arm (chemotherapy + endoscopic thermal ablation). However, the overall survival was similar regardless of the type of treatment and thus not statistically significant. Moreover, the study confirmed the safety of the EUS HybridTherm probe in patients with PDAC. As the study was underpowered, larger randomized trials are needed to investigate the role of EUS-guided localized ablation after induction with chemotherapy.

In conclusion, this Special Issue deepens our understanding of various aspects of endoscopic diagnosis and treatment for liver and pancreatic cancers. Since these tumors are characterized by a high incidence rate and aggressiveness, diagnostic tools with high accuracy and adequacy of sampled materials are vital to high quality patient care. Moreover, prompt individualized oncologic treatment with promising endoscopic techniques is a multidisciplinary field that deserves the efforts of endoscopists together with oncologists and surgeons [21].
